# Docosahexaenoic Acid Supplementation in Postnatal Growth Restricted Rats Does Not Normalize Lung Function or PPARγ Activity

**DOI:** 10.3390/biom15040551

**Published:** 2025-04-09

**Authors:** Adrienne J. Cohen, Wesley R. Chidester, Daniel T. Wray, Nicolette Jessen, Aimee Jones, Cheylah Bitsui, James Zhao, J. Alan Maschek, James E. Cox, Camilia R. Martin, Lisa A. Joss-Moore

**Affiliations:** 1 Department of Pediatrics, University of Utah, Salt Lake City, UT 84108, USAwesley.r.chidester@utah.edu (W.R.C.);; 2 Health Science Center Cores, University of Utah Health Sciences Center, Salt Lake City, UT 84108, USA; 3 Department of Biochemistry, University of Utah, Salt Lake City, UT 84108, USA; 4 Division of Neonatology, Weill Cornell Medicine, New York, NY 10065, USA

**Keywords:** lung development, growth restriction, bronchopulmonary dysplasia, DHA, PPARγ, alternative splicing

## Abstract

The development of BPD in preterm neonates is increased by poor growth and nutritional deficits. The involvement of the fatty acid DHA in the development of BPD has been a focus for over a decade. However, recent clinical trials show that isolated DHA supplementation may increase BPD in subgroups of preterm neonates. One explanation for poor lung outcomes in DHA-supplemented neonates is a disruption of global fatty acid profiles and increased expression of a dominant-negative splice variant of a key driver of lung development, PPARγ. We previously developed a rat model of postnatal growth restriction (PGR) in which pups have impaired lung function and altered PPARγ activity. Here, we use our PGR rat model to assess the effects of DHA supplementation on lung outcomes. We hypothesize that the PPARγ splice variant, PPARγΔ5, will be expressed in the rat lung, and that DHA supplementation of PGR rat pups will alter circulating lipid profiles, lung mechanics, and PPARγ variant expression. Our findings demonstrate that PPARγΔ5 is expressed in the developing rat lung and that DHA supplementation of PGR rat pups alters global circulating fatty-acid profiles and does not normalize PGR-induced impaired lung mechanics or PPARγ activity.

## 1. Introduction

Preterm birth and poor growth increase the risk of developing the chronic lung disease bronchopulmonary dysplasia (BPD). While the development of BPD in preterm neonates is multifactorial, poor growth during the pre- and postnatal periods, along with nutritional deficits contribute to the BPD lung phenotype of impaired alveolar formation. Important nutrients in the context of BPD and lung development include long-chain polyunsaturated fatty acids (LCPUFAs), particularly the long-chain omega 3 fatty acid docosahexaenoic acid (DHA) [1].

The involvement of DHA deficiency in the development of BPD, and potential protective effects of DHA supplementation in resolving lung outcomes in preterm neonates and in animal studies has been a focus for over a decade. In preterm infants born <30 weeks gestation, DHA decreases rapidly over the first week of life, and decreased DHA is associated with increased BPD [2]. Other studies conducted to assess the effects of DHA supplementation on neurodevelopment, with BPD as a secondary outcome, showed a positive impact of enteral DHA supplementation on BPD in a subset of preterm neonates [3]. The concept that DHA is beneficial to the developing lung, particularly in the context of pre- and postnatal growth restriction and neonatal lung injury, is also supported by animal studies [4,5,6,7]. As a result of the body of research supporting a role for DHA in the development of BPD, several clinical trials examining the effects of DHA supplementation of preterm infants on BPD rates and severity have been conducted. However, these trials show that supplemental DHA administration to preterm neonates may actually increase the incidence of BPD in subsets of neonates [8,9]. Given the need for, and potential consequences of, lipid supplementation of preterm neonates, a comprehensive understanding of the effects of lipid supplementation on the developing lung is essential.

One key lipid responsive molecular mediator of lung development is the transcriptional activator PPARγ. PPARγ is well established to have important functions in the lung and is necessary for the epithelial–mesenchymal interactions required for lung development and lung vascular integrity [10,11,12]. PPARγ is also essential in the lung response to injury, and injury effects can often be mitigated by PPARγ activation, which can be accomplished by DHA [4,13,14,15,16]. Like many transcription factors, PPARγ is subject to extensive alternative splicing. One splice variant of PPARγ, the PPARγ delta 5 (PPARγΔ5) variant, has been reported in adipose and kidney tissue, and functions as a dominant-negative protein isoform due to lack of a transactivation domain [17,18]. However, whether PPARγΔ5 is expressed in the lung, and whether its expression is altered by PGR and/or DHA supplementation is unknown.

In order to elucidate mechanistic effects of growth and DHA on lung outcomes, we use a rat model of postnatal growth restriction (PGR) following normal in utero growth [19]. We recently showed that prolonged PGR, without additional lung injury, impairs lung structure and function in the rat. In this model, the lung characteristics are similar to those of neonates with BPD, including increased alveolar wall thickness, decreased lung compliance, and increased tissue damping [19]. Here, we aim to determine the effects of DHA supplementation on lung outcomes in our previously described PGR rat model, as well as expression of PPARγ and the PPARγΔ5 splice variant. We hypothesize that the PPARγ splice variant, PPARγΔ5, will be expressed in the rat lung, and that DHA supplementation of PGR rat pups will alter circulating lipid profiles, lung mechanics, and PPARγ variant expression.

## 2. Materials and Methods

### 2.1. Rat Model of PGR

All animal procedures were reviewed and approved by the University of Utah Animal Care and Use Committee, following the NIH Guidelines for the Care and Use of Laboratory Animals. Timed pregnant Sprague Dawley rats (Charles River, Wilmington, MA, USA) were housed in a controlled environment with a 12 h light/dark cycle and unrestricted access to food and water. We employed the postnatal growth restriction (PGR) model as previously described by our group [19]. Briefly, at birth, pups were assigned to foster dams to establish litter sizes of either eight (control) or sixteen (PGR). Pups remained within their respective litters for the study duration. On postnatal day 21, animals were either euthanized for tissue and serum collection (decapitated under anesthesia with ketamine/xylazine at 80/12 mg/kg) or assigned to lung mechanics assessments. Each litter contributed one male and one female pup for experimental analyses.

### 2.2. DHA Supplementation

At delivery, rat dams from the PGR groups were randomized to receive either standard rat chow (6% fat from soybean oil, EnVIGO Teklad, Indianapolis, IN, USA) or rat chow supplemented with DHA at 0.01% of total fats (LoDHA) or DHA at 0.1% of total fats (HiDHA) (Nu-Chek-Prep, Inc., Elysian, MN, USA). The DHA chow was formulated by substituting the desired percentage of soybean oil with pure DHA. All rat chow formulations were isocaloric and equally tolerated by the dams. All dams had ad libitum access to chow and water during lactation.

### 2.3. Serum Fatty Acids

Serum fatty acid composition was analyzed via gas chromatography following transesterification, as previously described by our group [19,20]. Fatty acid methyl esters (FAMEs) were generated via transesterification using heptadecanoic acid as an internal standard for quantification. Analyses were performed on an Agilent 7890A gas chromatograph (Agilent Technologies, Santa Clara, CA, USA) equipped with a capillary column, enabling the detection of fatty acids with chain lengths ranging from 10 to 24 carbons. FAMEs were identified and quantified using the Supelco 37 FAME mix (Sigma-Aldrich, St. Louis, MO, USA), and data processing was conducted with OpenLAB Chromatography software (Agilent Technologies).

### 2.4. Lung Mechanics

Lung mechanics were assessed using the FlexiVent FX 2 system (SCIREQ, Montreal, QC, Canada) and analyzed with FlexiWare 7.2 software (Service Pack 2, Build 728; SCIREQ, Montreal, QC, Canada). On postnatal day 21, rat pups were anesthetized via intraperitoneal injection of ketamine (50 mg/kg) and xylazine (8 mg/kg). Following tracheostomy, a 16- or 18-gauge cannula was inserted, and animals were connected to the FlexiVent system. Ventilation was maintained at a rate of 150 breaths per minute with tidal volumes of 10 mL/kg and a positive end-expiratory pressure of 3 cm H_2_O. After a stabilization period of 3 min to establish consistent breathing patterns and confirm the absence of air leaks, vecuronium bromide (1 mg/kg, IP) was administered as a paralytic agent.

Lung function was evaluated using automated maneuvers, as previously described [21]. Inspiratory capacity (IC) and static compliance (Cst) were derived from quasistatic pressure–volume (PV) loops recorded over a pressure range of 3 to 30 cm H_2_O. To differentiate airway mechanics from parenchymal properties, the forced oscillation technique (FOT) was applied [21]. Respiratory impedance data were modeled using the constant phase equation to obtain Newtonian resistance (Rn), an index of airway narrowing, as well as tissue elastance (H) and tissue damping (G), which reflect alveolar stiffness and resistance, respectively. Tissue hysteresivity (η, G/H) was also calculated to assess viscoelastic properties of the lung.

### 2.5. Identification of PPARγΔ5 in the Lung

Lung RNA was extracted from lung tissue from control rats at birth, postnatal day 12, and postnatal day 21 and reverse transcribed as previously described [19]. Gene specific primers were used to amplify the region of the Pparγ transcript between exon 4 and exon 6. Primer sequences were forward (within exon 4), CGAGAAGGAGAAGCTGTTGG, and reverse (within exon 6), GCACGTGCTCTGTGACAATC. The PCR product was subject to gel electrophoresis using 2.2% agarose FlashGel^®^ Recovery Cassettes and measured (57022, Lonza Bioscience, Rockland, ME, USA). Resulting bands were excised and sequenced by the University of Utah Sequencing Core.

### 2.6. mRNA Transcript Levels

Real-time reverse transcriptase PCR was used as described by our group [19,22] to measure the abundance of lung Pparγ and of the PparγΔ5 splice variant. We used an assay-on-demand primer/probe set for Pparγ, which covers the exon boundary between exon 5 and 6 (Rn00440945_m1, ThermoFisher Scientific, Waltham, MA, USA). To measure levels of PparγΔ5, we used a custom primer/probe set spanning the exon 4–6 junction (forward, CGAGAAGGAGAAGCTGTTGG; reverse, GCGGTTGATTTGTCTGTTGT; probe, CCCTGGCAAAGCATTTGTAT). mRNA levels of PPARγ target gene, Perilipin 2 (Plin2) was also measured using the following Assay on Demand: Rn01399516_m1. For all measures, the comparative CT method was used, with GADPH as an internal control.

### 2.7. Protein Abundance

We utilized immunoblot to measure protein levels of PPARγ and PPARγΔ5. Proteins were separated NuPAGE™ Bis-Tris Midi Protein Gels, 4 to 12% (WG1403BOX, Invitrogen, Waltham, MA, USA). Following electrophoresis, protein was transferred using PVDF Transfer Stacks (IB34001, Invitrogen, Waltham, MA, USA) and dry-transfer with the iBlot™ 3 Western blot Transfer Device (IB31001, Invitrogen, Waltham, MA, USA). Total protein normalization was performed using No-Stain™ Protein Labeling Reagent according to manufacturer instructions (A44449, Invitrogen, Waltham, MA, USA). Membranes were probed using anti-PPARγ polyclonal antibody (16643-1-AP, Proteintech, Rosemont, IL, USA) in SuperSignal™ Western blot Enhancer. Membranes were incubated in SuperSignal™ West Pico PLUS Chemiluminescent Substrate according to manufacturer instructions (34580, Thermo Scientific, Waltham, MA, USA), and imaged using Universal settings of the iBright™ CL1500 Imaging System (A44114, Invitrogen, Waltham, MA, USA). Unedited and uncropped blots are available in Supplemental Data.

### 2.8. Statistics

Groups were the control receiving a regular diet (Control), PGR with a regular diet (PGR), PGR with 0.01% DHA diet (PGR + LoDHA), and PGR with 0.1% DHA diet (PGR + HiDHA). Males and females were treated as separate groups. A one-way analysis of variance (ANOVA) with Fisher’s post hoc protected least-significance difference was used to determine differences between groups. Statistical significance was defined as *p* < 0.05.

## 3. Results

### 3.1. Rat Model of PGR and DHA Supplementation

As we previously reported, our model of PGR resulted in a significant decrease in weight at day 21 in both males and females compared to control (Table 1). PGR with a regular diet resulted in a 33% decrease in body weight in males. The addition of DHA supplementation in male rat pups did not improve weight gain in PGR, with PGR + LoDHA and PGR + HiDHA rat pups weighing 38% and 27% less than male controls, respectively. Similarly, in females, PGR resulted in a 34% reduction in body weight in the regular diet group, and a 41% and 28% reduction in body weight with PGR + LoDHA and PGR + HiDHA, respectively.

### 3.2. Serum Fatty Acids

Both PGR and DHA supplementation significantly affected serum fatty acid levels (Table 2). PGR with a regular diet altered serum levels of several fatty acids in male rat pups. In male rat pups, PGR on a regular diet decreased serum levels of stearic acid (18:0) by 40%, oleic acid (18:1) by 43%, linoleic acid (18:2n6) by 40%, and DHA (22:6n3) by 33% compared to controls. PGR also increased the ARA/DHA ratio by 40% in male rat pups. In contrast, in female rat pups, PGR on a regular diet did not alter levels of any serum fatty acid.

Consistent with the experimental design, serum DHA was increased by maternal DHA supplementation in PGR rat pups. In male PGR rat pups, the LoDHA diet increased serum DHA by 140% compared to PGR on a regular diet, while the HiDHA diet increased serum DHA by 520% compared to PGR on a regular diet and 315% compared to controls. In female PGR rat pups, the HiDHA diet increased serum DHA by 370% compared to PGR on a regular diet and 360% compared to controls.

DHA supplementation of PGR rat pups also significantly altered serum levels of other fatty acids. In male PGR rat pups, the Lodha diet normalized levels of oleic acid (18:1), linoleic acid (18:2n6), and the ARA/DHA ratio, while serum levels of palmitoleic acid (16:1) decreased by 60% compared to controls, and palmitic acid (16:0) increased by 45% compared to controls. In female PGR rat pups, the Lodha diet also decreased levels of palmitoleic acid (16:1) by 75% compared to controls. In male PGR rat pups, the HiDHA diet increased serum levels of ARA by 77% compared to controls while also decreasing the LA/DHA ratio by 75% and the ARA/DHA ratio by 56% compared to controls. Similarly, in female PGR rat pups, the HiDHA diet decreased the LA/DHA ratio by 75% and the ARA/DHA ratio by 55%.

### 3.3. Lung Mechanics

Using closed chest pressure–volume loops to determine quasi-static lung compliance and the forced oscillation technique to differentiate between central and peripheral lung tissue, we measured lung mechanics in all four groups (control, PGR, PGR + LoDHA, PGR + HiDHA).

**Figure 1:**
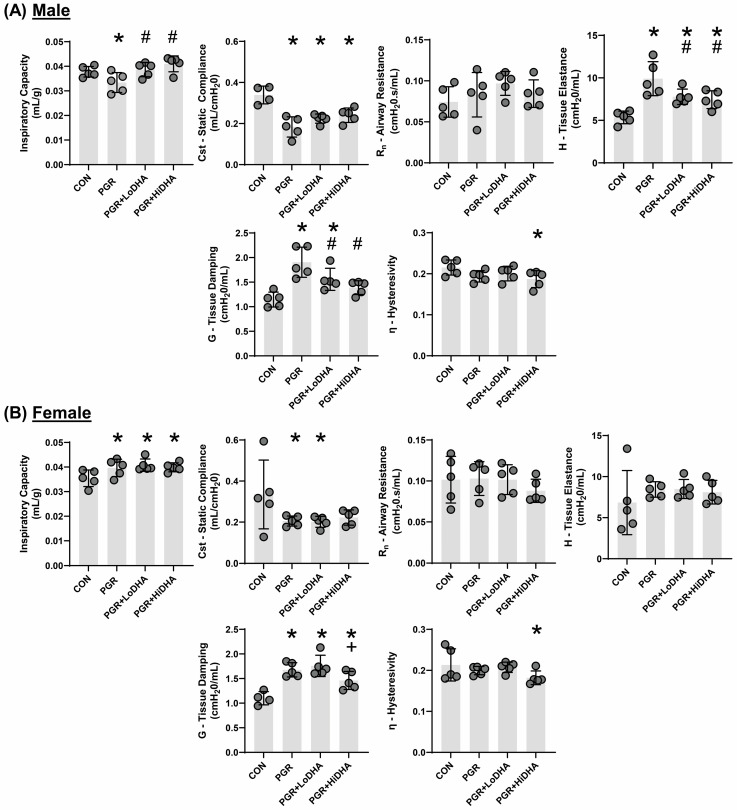
PGR with and without DHA supplementation alters rat lung mechanics. (**A**) Lung function measurements in male and (**B**) female rat pups. Data points are individual rat pups, bars are mean with SD. * *p* ≤ 0.05 compared to sex-matched control, # *p* ≤ 0.05 compared to PGR, + *p* ≤ 0.05 compared to PGR + LoDHA.

Similar to our previous report [19], PGR altered lung mechanics in male and female rat pups. In male rat pups, PGR on a regular diet decreased inspiratory capacity by 12%, decreased static compliance by 46%, and increased tissue elastance by 85% and tissue damping by 66% compared to controls (Figure 1A). In female rat pups, PGR increased inspiratory capacity by 11%, decreased static compliance by 39%, and increased tissue damping by 52% compared to controls (Figure 1B).

DHA supplementation of PGR rat pups did not restore lung function to that of controls in either sex. In male PGR rat pups, while inspiratory capacity was normalized, static lung compliance remained reduced (34% and 30% decrease compared to controls with LoDHA and Hi DHA diets, respectively), and tissue elastance remained increased (45% and 38% increase compared to controls with LoDHA and HiDHA diets, respectively). Tissue damping remained increased male PGR rat pups with LoDHA diet (35% increase relative to control), but was normalized with the HiDHA diet. Additionally, the HiDHA diet decreased lung hysterestivity by 14% compared to the control (Figure 1A). In female PGR rat pups, inspiratory capacity remained increased (15% and 12% increase compared to controls with LoDHA and Hi DHA diets, respectively) and static lung compliance remained reduced with the LoDHA diet (40% compared to controls) but was normalized with the HiDHA diet. Tissue damping remained increased female PGR rat pups on both diets (59% and 32% increase compared to controls with LoDHA and HiDHA diets, respectively). Similarly to male PGR rat pups, in female PGR rat pups, the HiDHA diet decreased lung hysterestivity by 15% compared to the control (Figure 1B).

Overall, the effects of PGR on a regular diet were consistent with our previously published data [19]. The addition of DHA to PGR rat pups resulted in some sex-divergent improvements in lung function. Improvements included, in male rat pups, normalized IC, improved tissue elastance, and tissue damping, with normalization at high DHA levels. In female rat pups, static compliance was normalized at the high DHA dose. However, significant lung function deficits remain in both male and female PGR rat pups on a DHA diet.

### 3.4. PPARγΔ5 in the Lung

**Figure 2:**
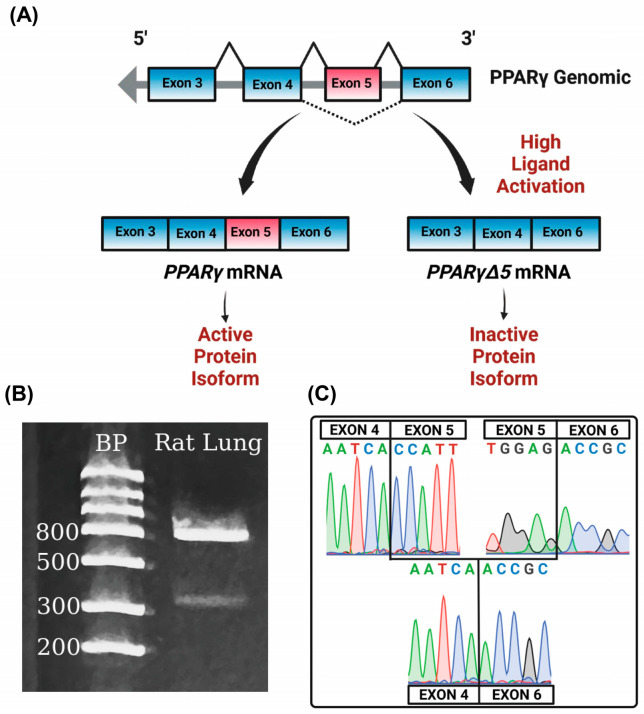
*PparγΔ5* splice variant is expressed in the lung. (**A**) Schematic of *Pparγ* alternative splicing, (**B**) gel electrophoresis of PCR across exon 4 to 6. Products are full-length *Pparγ* (850 bp) and *PparγΔ5* splice variant in lung tissue (450 bp), (**C**) corresponding sequencing results of the upper and lower bands.

To determine whether *PparγΔ5* is expressed in the lung, we performed PCR on cDNA isolated from rat lungs, using primers contained within exon 4 (forward) and exon 6 (reverse). Gel electrophoresis of our PCR product produced two bands, one measuring approximately 850 bp and the other measuring approximately 450 bp, consistent with the estimated molecular weights of the targeted amplicons of full-length *Pparγ* and the *PparγΔ5* splice variant, respectively (Figure 2B). Sequencing confirmed that the 850 bp band amplified *Pparγ* containing exon 5, and the 450 bp band amplified the *PparγΔ5* variant with exon four spliced directly to exon 6 (Figure 2C).

### 3.5. PPARγ mRNA and Protein Quantification

**Figure 3:**
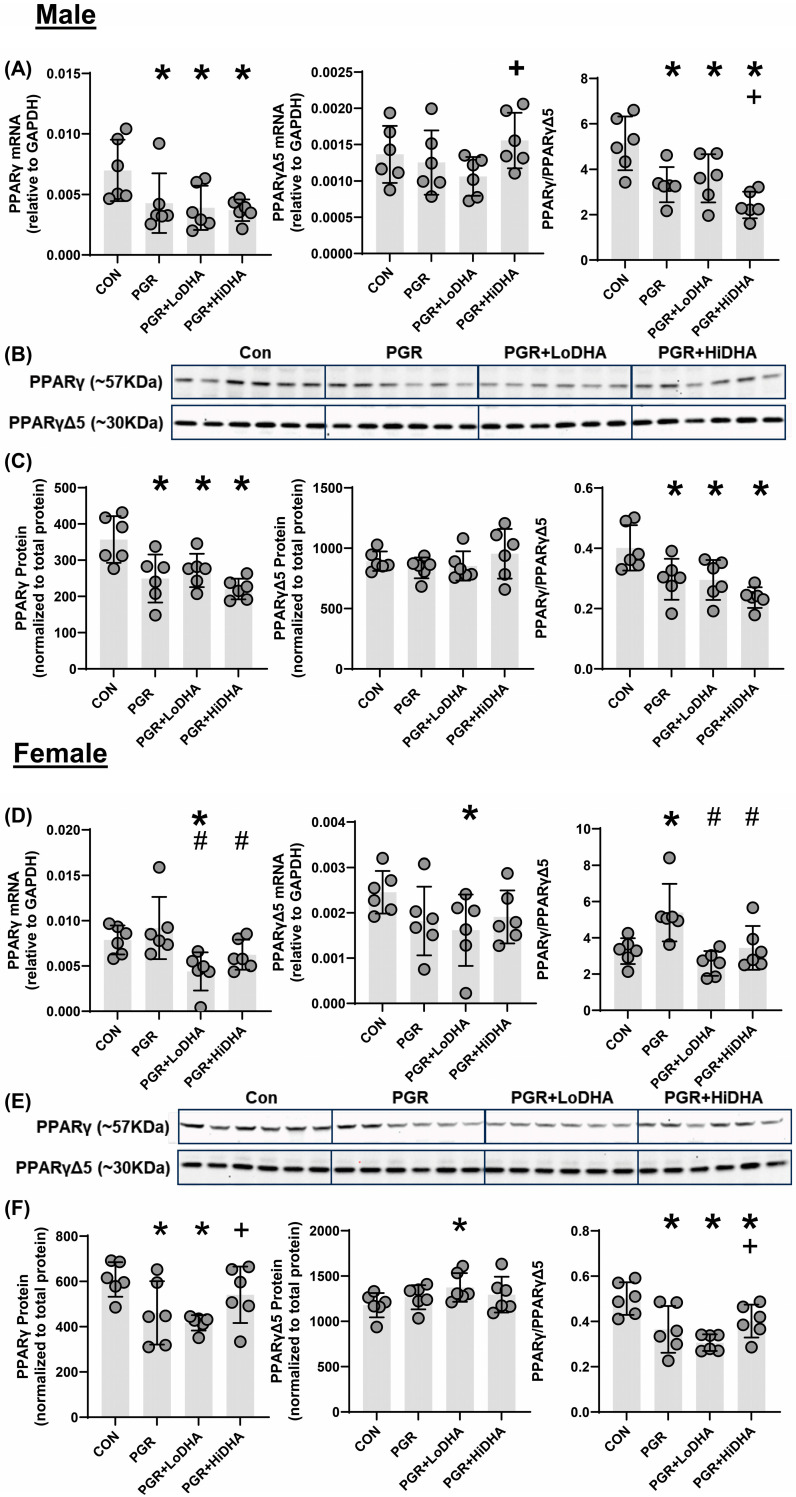
PGR with and without DHA supplementation alters expression of PPARγ mRNA variants and protein isoforms in the lung. (**A**) Male rat lung Pparγ, mRNA PparγΔ5 mRNA, and Pparγ/PparγΔ5 mRNA ratio. (**B**) Western blot images of male rat lung PPARγ and PPARγΔ5, (**C**) Male rat lung protein levels of PPARγ and PPARγΔ5 and the resulting ratio (normalized to total protein). (**D**) Female rat lung Pparγ mRNA, PparγΔ5 mRNA, and Pparγ/PparγΔ5 mRNA ratio. (**E**) Western blot images of female rat lung PPARγ and PPARγΔ5. (**F**) Female rat lung protein levels of PPARγ and PPARγΔ5 and the resulting ratio (normalized to total protein). Data points are individual rat pups, bars are mean with SD. * *p* ≤ 0.05 compared to sex-matched control, # *p* ≤ 0.05 compared to PGR, + *p* ≤ 0.05 compared to PGR + LoDHA.

We quantified levels of *Pparγ* and *PparγΔ5* mRNA and PPARγ and PPARγΔ5 protein in all study groups. In male rat lung, PGR with a regular diet decreased *Pparγ* mRNA by 39% compared to control but did not alter *PparγΔ5* mRNA (Figure 3A). The *Pparγ/PparγΔ5* ratio was decreased in male the PGR rat lung on a regular diet by 35% relative to control (Figure 3A). In male rat lung, PGR with a regular diet decreased PPARγ protein levels by 30% compared to control, without affecting protein levels of PPARγΔ5. Similarly to mRNA findings, in the male rat lung, PGR with a regular diet decreased the PPARγ/PPARγΔ5 ratio by 26% compared to control (Figure 3B,C). In the female rat lung, PGR with a regular diet did not affect mRNA levels of *Pparγ* or *PparγΔ5* (Figure 3D). However, the resulting *Pparγ/PparγΔ5* ratio was increased in the female PGR rat lung on a regular diet by 65% compared to control (Figure 3D). In the female rat lung, PGR with a regular diet decreased PPARγ protein levels by 24% compared to control, without affecting protein levels of PPARγΔ5. In a female rat lung, PGR with a regular diet decreased the PPARγ/PPARγΔ5 ratio by 27% compared to control (Figure 3E,F).

In male PGR rat lung, *Pparγ* mRNA levels remained decreased with both DHA diets (44% and 46% compared to control for LoDHA and HiDHA, respectively), and *PparγΔ5* mRNA remained unchanged (Figure 3A). Similarly, in male PGR rat lung, PPARγ protein levels remained decreased with both DHA diets (24% and 38% compared to control for LoDHA and HiDHA, respectively), and PPARγΔ5 protein levels were unaffected (Figure 3B,C). The resulting PPARγ/PPARγΔ5 protein ratio also remained reduced in male PGR rat lung with both DHA diets (27% and 41% compared to control for LoDHA and HiDHA, respectively).

In female PGR rat lung, *Pparγ* and *PparγΔ5* mRNA levels decreased with the LoDHA diet (44% and 34%, respectively, compared to control) (Figure 3D). In the female PGR rat lung, PPARγ protein levels decreased with the LoDHA diet by 32% compared to control, while PPARγΔ5 protein levels increased by 17% relative to the control (Figure 3E,F). The resulting PPARγ/PPARγΔ5 protein ratio, however, remained reduced in female PGR rat lung with both DHA diets (39% and 20% compared to control for LoDHA and HiDHA, respectively).

Overall, DHA supplementation did not normalize any PPARγ measure in the male PGR rat lung but did normalize PPARγ mRNA and PPARγ protein at a high dose in female rat pups. However, the PPARγ/PPARγΔ5 ratio was not normalized in the female rat lung.

### 3.6. mRNA of Downstream PPARγ Target Gene Plin2

**Figure 4:**
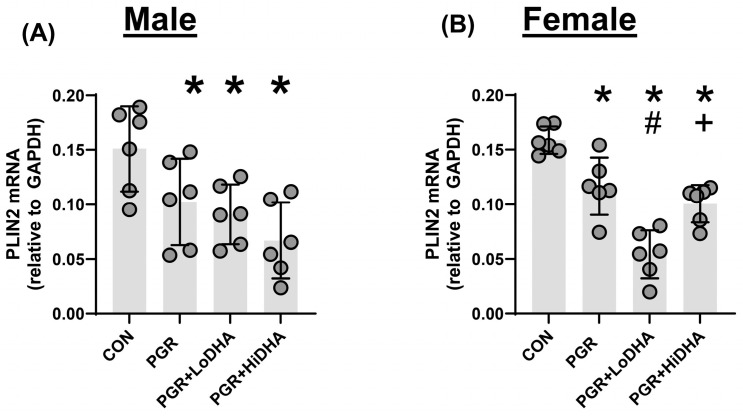
PGR and DHA supplementation decreases the expression of PPARγ target gene Plin2. (**A**) Male rat lung Plin2 mRNA levels. (**B**) Female rat lung Plin2 mRNA levels. * *p* ≤ 0.05 compared to sex-matched control, # *p* ≤ 0.05 compared to PGR, + *p* ≤ 0.05 compared to PGR + LoDHA.

To assess PPARγ activity, we measured mRNA levels of the PPARγ target gene Perilipin-2 (*Plin2*). In the male rat lung, PGR with a regular diet decreased *Plin2* mRNA levels by 32% compared to controls (Figure 4A). In the female rat lung, PGR with a regular diet decreased *Plin2* mRNA levels by 27% compared to controls (Figure 4B). In the male rat PGR lung, *Plin2* mRNA remained decreased with both DHA diets (40% and 56% compared to control for LoDHA and HiDHA, respectively) (Figure 4A). In the female rat PGR lung, *Plin2* mRNA also remained decreased with both DHA diets (66% and 37% compared to control for LoDHA and HiDHA, respectively) (Figure 4B).

Overall, the addition of DHA to PGR rat pups did not normalize lung mRNA levels of the PPARγ target gene Plin2 in either male or female rat lung.

## 4. Discussion

The development of bronchopulmonary dysplasia is multifactorial, and the molecular drivers and prevention of this disease remain poorly understood. Given the confounding data demonstrating the importance of DHA in lung development and prevention of BPD, combined with the recent clinical trials demonstrating that DHA supplementation of preterm neonates may increase BPD, a comprehensive understanding of the molecular effects of DHA supplementation in the developing lung is required. Using a combined model of postnatal growth restriction and DHA supplementation, we show that postnatal growth restriction in the absence of in utero growth restriction, or additional lung injury, alters circulating fatty acids and impairs lung mechanics in rats. We also show that these effects are not normalized by DHA supplementation. We also demonstrate, for the first time, that a dominant negative splice variant of PPARγ, a key regulator of lung development, is expressed in the developing lung. We show that PPARγ activity is decreased in PGR and not normalized with DHA supplementation. Collectively, our data support the view that postnatal DHA supplementation does not normalize lung outcomes in PGR.

The negative impact of nutritional deficits in the development of BPD is well accepted [1,23,24,25]. In utero growth restriction and lower birth weight are associated with higher rates of BPD and worse lung outcomes in human neonates [26,27,28]. Even when prenatal growth is not impaired, postnatal growth deficits have been linked to elevated BPD risk and adverse pulmonary outcomes [29,30,31]. Postnatally, poor nutrition in human preterm infants occurs secondary to feeding intolerance and clinically indicated feeding volume restriction. In this study, we focused on PGR in the context of normal in utero growth and the absence of additional lung injury, such as hyperoxia. We found that PGR on a regular diet significantly impaired lung function parameters in both male and female rat pups. The addition of DHA to PGR pups resulted in improvements in some lung function parameters, and no change in others. In addition, these effects were sex-divergent. For example, in male PGR rats, DHA supplementation normalized tissue damping without improving static compliance, suggesting that DHA had beneficial effects on dynamic aspects of lung function without changing the overall lung structure or elasticity at total lung capacity. One possible mechanism for improved dynamic lung function by DHA is via effects on surfactant production, as improved surfactant production following PPARγ activation has been demonstrated in other models of lung injury [32,33]. In contrast, the lack of improvement in static lung compliance by DHA in PGR rats may reflect a structural alteration in the lung caused by PGR, which is not improved by DHA. We previously demonstrated that PGR on a regular diet impairs alveolar formation with a concomitant increase in static compliance, parameters consistent with failed alveolar formation in BPD [19,34]. Examination of DHA administration to PGR rat pups at an earlier postnatal time point, i.e., during the saccular-to-alveolar transition, is an important next step in determining whether the timing of DHA administration increases favorable outcomes. Sex-divergent outcomes in lung parameters in human neonates are also well accepted, with males generally at a risk of higher incidence and greater severity of BPD. A complete understanding of sex-divergent outcomes in human neonates remains elusive [35]. However, studies suggest that chromosomal sex, as opposed to gonadal sex, is likely a driving force [36].

Recent clinical trials examining the effects of DHA supplementation of preterm neonates used either direct enteral supplementation of DHA to preterm neonates (N-3 fatty acids for improvement in respiratory outcomes (N3RO) trial), or DHA administration via maternal DHA supplementation and the provision of breastmilk to preterm neonates (The Maternal Omega-3 Supplementation to Reduce Bronchopulmonary Dysplasia in Very Preterm Infants (MOBYDIck) trial) [8,9]. The N3RO trial ultimately concluded that neonatal DHA supplementation did not improve the overall incidence of BPD and may increase the incidence of BPD in neonates born at less than 27 weeks. Considering these results from the N3RO trial, an early interim analysis of the MOBYDIck trial was performed. The analysis favored the placebo over DHA in BPD-free survival, and the study was terminated due to concern for potential harm to future participants. These outcomes were surprising given the dearth of information demonstrating the importance of DHA in lung development and response to injury, and several rationales for these outcomes and currently being explored. One potential contributor to the negative effects of isolated DHA supplementation is the effect of increased DHA on other circulating fatty acids, including the omega-6 fatty acid ARA.

In the N3RO trial, blood levels of ARA were decreased in the DHA group, as was the ARA/DHA ratio [8]. In the MOBYDIck trial, blood lipids were not assessed. However, the average lipid composition of the maternal milk used in the trial was analyzed at two weeks [37]. Findings from this analysis showed that while maternal DHA supplementation increased milk DHA levels, supplementation decreased the ARA/DHA ratio [37]. The potential importance of including ARA in supplementation is highlighted by other studies that used supplementation with a combination of ARA and DHA. Collectively, the studies that utilized a combination of ARA and DHA (at a 2:1 ratio) did not report negative effects on BPD outcomes [38,39]. The changes to the ω3-to-ω6 ratio and to arachidonic acid levels are especially notable, given the role ARA plays in modulating the inflammatory cascade. In our study, we examined two doses of DHA administered via the maternal diet. Our low-dose DHA diet contained 0.01% DHA, which increased serum DHA levels by approximately 1% of total lipids in male and female PGR pups compared to those on a regular diet. Our low DHA diet increased serum DHA to a similar degree as the 60 mg/kg dose of DHA used in the N3RO trial [8]. Our high dose DHA diet contained 0.1% DHA, which increased serum DHA by approximately 6.5% of total fatty acids. In our study, DHA supplementation also significantly reduced the ARA/DHA ratio in both male and female rat pups, again with the low dose DHA diet similar to that of the N3RO trial.

An important consideration in the context of DHA supplementation, and disturbed circulating fatty acid profiles during lung development, is the transcriptional activator, PPARγ. PPARγ is required for appropriate lung development, particularly the formation of alveoli. Conditional knockout of PPARγ in mouse airway epithelial cells leads to airspace enlargement and disruption of epithelial–mesenchymal interactions [10]. PPARγ activation also promotes myofibroblast differentiation to a lipid-laden lipofibroblast phenotype in the developing lung, characterized by high triglyceride levels, which has a significant role in lung maturation, including alveolar maturation [40,41,42]. We and others have previously shown that neonatal rat pups exposed to fetal growth restriction or postnatal hyperoxia have decreased PPARγ expression and impaired alveolar formation. In both cases, lung phenotypes are reversed by PPARγ activation [4,16]. An important consideration, however, is that to achieve greater regulation, transcriptional activators also undergo alternative splicing and express dominant negative variants [43].

PPARγ produces a dominant negative isoform, PPARγΔ5, via the alternative splicing of exon 5 [17]. Studies in adipose tissue have shown that expression of PPARγΔ5 is increased by ligand activation of PPARγ. The protein isoform produced by alternative splicing of PPARγ exon 5 results in a premature stop codon and truncated protein isoform lacking the ligand binding domain and one transactivation domain [17]. The truncation also results in the removal of a ubiquitin binding domain, thus potentially making the truncated protein more stable than the native isoform [44]. In adipose tissue, a decrease in the cellular PPARγ/PPARγΔ5 ratio reduces transcription of downstream target genes. In this study, we demonstrate, for the first time, that the PPARγΔ5 variant is expressed in the rat lung under control conditions. Given that DHA can act as a ligand for PPARγ, we considered whether supplemental DHA would increase the expression of PPARγΔ5 in the lung [45]. Our data show that supplemental DHA has a limited effect on the levels of PPARγΔ5 mRNA or protein in the rat lung. However, given that the PPARγ/PPARγΔ5 ratio conveys overall functional potential, this may be a more appropriate metric. We demonstrated that PGR alone decreases the PPARγ/PPARγΔ5 ratio in the lung in both male and female rat pups, and that this decrease is associated with a decrease in the mRNA levels of the target gene Plin2 (also known as adipose differentiation-related protein), another critical player in lung development [46]. Reduced PPARγ activity and expression of Plin2 are both associated with impaired lung development and the structural changes consistent with the altered lung function measures we detected. The addition of DHA to PGR rats in this study did not completely normalize PGR-induced impaired lung function, nor did the diets significantly alter the PGR-induced reduction in PPARγ activity. An important caveat for our study is the timing of rat lung development. At our study time point, postnatal day 21, the rat lung has completed bulk alveolar formation. It will be important in subsequent studies to assess the effects of DHA on the rat lung during the transition from the saccular to alveolar stages. Also important will be studies aimed at determining the precise mechanisms by which PGR decreases the PPARγ/PPARγΔ5 ratio, and the cause-and-effect consequences of this change on lung development.

Our study is not without limitations. In this study, we only examined circulating fatty acid profiles. An understanding of how PGR and DHA supplementation affect intrapulmonary fatty acid profiles is needed. An advantage of understanding intrapulmonary fatty acid profiles and circulating fatty acid profiles in the same system is the potential for the identification of fatty acid biomarkers that may be useful in identifying neonates with detrimental fatty acid complements. It will also be important in future studies to determine the expression of PPARγΔ5 in human lung cells. Other studies have identified the expression of PPARγΔ5 in human tissue [17], but to date, not in the human lung. We also have not explored the origins of the sex differences observed in our study. Several potential interactions may contribute to the differences in sex outcomes in the lung, a topic that should be addressed in future studies. Lastly, an analysis assessing correlations between PPARγ activity and lung function parameters would be informative to understand the overall effects of DHA supplementation of PGR rat pups. However, as a correlation analysis requires both variables to be assed in the same experimental subject, we were not able to perform this analysis. Because the protocol for lung function experiments requires administration of vecuronium bromide to the rats, we opted not to collect lung tissue for molecular experiments following the function studies. Therefore, our molecular measurements and our lung function experiments were performed in different rats and, thus, not appropriate for correlation analysis.

## 5. Conclusions

In conclusion, we demonstrate that the novel splice variant of PPARγ, PPARγΔ5, is expressed in the lung, and that PGR reduces the PPARγ/PPARγΔ5 ratio in association with impaired lung mechanics. DHA supplementation at and beyond clinically relevant doses improved some lung functional and molecular parameters. However, neither dose of DHA normalized all functional or molecular profiles of PGR rat pups. DHA supplementation did, however, disturb circulating fatty acid profiles for other LCPUFAs. Ongoing examination of the timing of DHA supplementation in the developing rat lung in the context of PGR is an important next step.

## Figures and Tables

**Table 1:** Rat body weight at study time, postnatal day 21.

	Control	PGR	PGR + LoDHA	PGR + HiDHA
Weight, g ± SD				
Male	53.4 ± 3.3	35.7 ± 4.5 *	33.0 ± 2.7 *	39.2 ± 3.5 *
Female	52.2 ± 3.8	34.6 ± 4.6 *	31.2 ± 3.6 *	37.6 ± 2.8 *

* *p* < 0.05 compared to control.

**Table 2:** Serum Fatty acids.

	Male					Female			
Fatty Acid (µg/mL ± SD)	Control	PGR	PGR +LoDHA	PGR +HiDHA		Control	PGR	PGR +LoDHA	PGR +HiDHA
Saturated									
Palmitic Acid (16:0)	537 ± 36	485 ± 142	775 ± 192 *^†^	699 ± 91 ^†^		633 ± 117	674 ± 133	676 ± 51	700 ± 81
Stearic Acid (18:0)	458 ± 162	268 ± 106 *	436 ± 113	352 ± 28		402 ± 158	477 ± 139	376 ± 60	380 ± 65
Monounsaturated									
Palmitoleic Acid (16:1)	34 ± 8	28 ± 7	14 ± 5 *^†^	18 ± 3 *^†^		44 ± 19	45 ± 20	11 ± 2 *^†^	21 ± 2 *^†^
Oleic Acid (18:1)	421 ± 171	240 ± 89 *	356 ± 94	392 ± 89		475 ± 182	561 ± 198	321 ± 62 ^†^	386 ± 46
ω-6									
Linoleic Acid (18:2n6)	1068 ± 320	642 ± 228 *	1261 ± 192 ^†^	1346 ± 261 ^†^		1047 ± 350	1258 ± 224	1135 ± 181	1191 ± 226
Arachidonic Acid (20:4n6)	1335 ± 439	1187 ± 972	2153 ± 445 ^†^	2367 ± 226 *^†^		1337 ± 498	1631± 658	1835 ± 542	2239 ± 508
ω-3									
Docosahexaenoic Acid (22:6n3)	142 ± 31	95 ± 44 *	228 ± 70 ^†^	589 ± 123 *^†‡^		145 ± 50	143 ± 62.0	175 ± 46	524 ± 96 *^†‡^
LA/DHA	8 ± 3	10 ± 6	6 ± 2	2 ± 0.1 *^†‡^		8 ± 2	10 ± 4	7 ± 2	2 ± 0.2 *^†‡^
ARA/DHA	9 ± 1	12 ± 2 *	10 ± 1 ^†^	4 ± 1 *^†‡^		9 ± 1	12 ± 4	10 ± 1	4 ± 0.3 *^†‡^

* *p* ≤ 0.05 compared to sex-matched control, ^†^ *p* ≤ 0.05 compared to sex-matched PGR with regular diet, ^‡^ *p* ≤ 0.05 compared to sex-matched PGR with low-dose DHA diet.

## Data Availability

The raw data supporting the conclusions of this article will be made available by the authors on request.

## Supplementary Materials

The following supporting information can be downloaded at: https://www.mdpi.com/article/10.3390/biom15040551/s1, Western Blot uncropped images.

## Abbreviations

The following abbreviations are used in this manuscript:

BPD	Bronchopulmonary Dysplasia
DHA	Docosahexaenoic Acid
PPARγ	Peroxisome Proliferator Activated Receptor gamma
PGR	Postnatal Growth Restriction
ARA	Arachidonic Acid
PLIN2	Perilipin 2

## References

[1] Poindexter B.B., Martin C.R. (2015). Impact of Nutrition on Bronchopulmonary Dysplasia. Clin. Perinatol..

[2] Martin C.R., DaSilva D.A., Cluette-Brown J.E., DiMonda C., Hamill A., Bhutta A.Q., Coronel E., Wilschanski M., Stephens A.J., Driscoll D.F. (2011). Decreased postnatal docosahexaenoic and arachidonic acid blood levels in premature infants are associated with neonatal morbidities. J. Pediatr..

[3] Makrides M., Gibson R.A., McPhee A.J., Collins C.T., Davis P.G., Doyle L.W., Simmer K., Colditz P.B., Morris S., Smithers L.G. (2009). Neurodevelopmental Outcomes of Preterm Infants Fed High-Dose Docosahexaenoic Acid. JAMA.

[4] Joss-Moore L.A., Wang Y., Baack M.L., Yao J., Norris A.W., Yu X., Callaway C.W., McKnight R.A., Albertine K.H., Lane R.H. (2010). IUGR decreases PPARγ and SETD8 Expression in neonatal rat lung and these effects are ameliorated by maternal DHA supplementation. Early Hum. Dev..

[5] Rogers L.K., Valentine C.J., Pennell M., Velten M., Britt R.D., Dingess K., Zhao X., Welty S.E., Tipple T.E. (2011). Maternal docosahexaenoic acid supplementation decreases lung inflammation in hyperoxia-exposed newborn mice. J. Nutr..

[6] Ali M., Heyob K.M., Velten M., Tipple T.E., Rogers L.K. (2015). DHA suppresses chronic apoptosis in the lung caused by perinatal inflammation. Am. J. Physiol. Cell. Mol. Physiol..

[7] Velten M., Britt R.D., Heyob K.M., Tipple T.E., Rogers L.K. (2014). Maternal dietary docosahexaenoic acid supplementation attenuates fetal growth restriction and enhances pulmonary function in a newborn mouse model of perinatal inflammation. J. Nutr..

[8] Collins C.T., Makrides M., McPhee A.J., Sullivan T.R., Davis P.G., Thio M., Simmer K., Rajadurai V.S., Travadi J., Berry M.J. (2017). Docosahexaenoic Acid and Bronchopulmonary Dysplasia in Preterm Infants. N. Engl. J. Med..

[9] Marc I., Piedboeuf B., Lacaze-Masmonteil T., Fraser W., Mâsse B., Mohamed I., Qureshi M., Afifi J., Lemyre B., Caouette G. (2020). Effect of Maternal Docosahexaenoic Acid Supplementation on Bronchopulmonary Dysplasia-Free Survival in Breastfed Preterm Infants: A Randomized Clinical Trial. JAMA.

[10] Simon D.M., Arikan M.C., Srisuma S., Bhattacharya S., Tsai L.W., Ingenito E.P., Gonzalez F., Shapiro S.D., Mariani T.J. (2006). Epithelial cell PPARγ contributes to normal lung maturation. FASEB J..

[11] Cerny L., Torday J.S., Rehan V.K. (2008). Prevention and treatment of bronchopulmonary dysplasia: Contemporary status and future outlook. Lung.

[12] Gien J., Tseng N., Seedorf G., Roe G., Abman S.H. (2014). Abman Peroxisome proliferator activated receptor-gamma-Rho-kinase interactions contribute to vascular remodeling after chronic intrauterine pulmonary hypertension. Am. J. Physiol. Lung Cell Mol. Physiol..

[13] Kulkarni A.A., Woeller C.F., Thatcher T.H., Ramon S., Phipps R.P., Sime P.J. (2012). Sime Emerging PPARgamma-Independent Role of PPARgamma Ligands in Lung Diseases. PPAR Res..

[14] Hagood J.S. (2014). Beyond the genome: Epigenetic mechanisms in lung remodeling. Physiology.

[15] Morales E., Sakurai R., Husain S., Paek D., Gong M., Ibe B., Li Y., Husain M., Torday J.S., Rehan V.K. (2014). Nebulized PPARγ agonists: A novel approach to augment neonatal lung maturation and injury repair in rats. Pediatr. Res..

[16] Rehan V.K., Wang Y., Patel S., Santos J., Torday J.S. (2006). Rosiglitazone, a peroxisome proliferator-activated receptor-γ agonist, prevents hyperoxia-induced neonatal rat lung injury in vivo. Pediatr. Pulmonol..

[17] Aprile M., Cataldi S., Ambrosio M.R., D’esposito V., Lim K., Dietrich A., Blüher M., Savage D.B., Formisano P., Ciccodicola A. (2018). PPARγΔ5, a Naturally Occurring Dominant-Negative Splice Isoform, Impairs PPARγ Function and Adipocyte Differentiation. Cell Rep..

[18] Shi C.-Y., Xu J.-J., Li C., Yu J.-L., Wu Y.-T., Huang H.-F. (2022). A PPARG Splice Variant in Granulosa Cells Is Associated with Polycystic Ovary Syndrome. J. Clin. Med..

[19] Zhao J., Ballard C., Cohen A.J., Ringham B., Zhao B., Wang H., Zuspan K., Rebentisch A., Locklear B.A., Dahl M. (2025). Postnatal growth restriction impairs rat lung structure and function. Anat. Rec..

[20] Weinheimer C., Wang H., Comstock J.M., Singh P., Wang Z., Locklear B.A., Goodwin K.L., Maschek J.A., Cox J.E., Baack M.L. (2020). Maternal Tobacco Smoke Exposure Causes Sex-Divergent Changes in Placental Lipid Metabolism in the Rat. Reprod. Sci..

[21] McGovern T.K., Robichaud A., Fereydoonzad L., Schuessler T.F., Martin J.G. (2013). Evaluation of Respiratory System Mechanics in Mice using the Forced Oscillation Technique. J. Vis. Exp..

[22] Joss-Moore L., Carroll T., Yang Y., Fitzhugh M., Metcalfe D., Oman J., Hale M., Dong L., Wang Z.-M., Yu X. (2013). Intrauterine growth restriction transiently delays alveolar formation and disrupts retinoic acid receptor expression in the lung of female rat pups. Pediatr. Res..

[23] Frank L., Sosenko I.R. (1988). Undernutrition as a major contributing factor in the pathogenesis of bronchopulmonary dysplasia. Am. Rev. Respir. Dis..

[24] Ehrenkranz R.A., Das A., Wrage L.A., Poindexter B.B., Higgins R.D., Stoll B.J., Oh W. (2011). Early nutrition mediates the influence of severity of illness on extremely LBW infants. Pediatr. Res..

[25] Morrow L.A., Wagner B.D., Ingram D.A., Poindexter B.B., Schibler K., Cotten C.M., Dagle J., Sontag M.K., Mourani P.M., Abman S.H. (2017). Antenatal Determinants of Bronchopulmonary Dysplasia and Late Respiratory Disease in Preterm Infants. Am. J. Respir. Crit. Care Med..

[26] Reiss I., Landmann E., Heckmann M., Misselwitz B., Gortner L. (2003). Increased risk of bronchopulmonary dysplasia and increased mortality in very preterm infants being small for gestational age. Arch. Gynecol. Obstet..

[27] Bose C., Van Marter L.J., Laughon M., O’Shea T.M., Allred E.N., Karna P., Ehrenkranz R.A., Boggess K., Leviton A. (2009). Fetal growth restriction and chronic lung disease among infants born before the 28th week of gestation. Pediatrics.

[28] Ambalavanan N., Van Meurs K.P., Perritt R., Carlo W.A., Ehrenkranz R.A., Stevenson D.K., Lemons J.A., Poole W.K., Higgins R.D. (2008). Predictors of death or bronchopulmonary dysplasia in preterm infants with respiratory failure. J. Perinatol..

[29] Ehrenkranz R.A., Dusick A.M., Vohr B.R., Wright L.L., Wrage L.A., Poole W.K. (2006). Growth in the neonatal intensive care unit influences neurodevelopmental and growth outcomes of extremely low birth weight infants. Pediatrics.

[30] Khan M.A., Kuzma-O’Reilly B., Brodsky N.L., Bhandari V. (2006). Site-specific characteristics of infants developing bronchopulmonary dysplasia. J. Perinatol..

[31] Figueras-Aloy J., Palet-Trujols C., Matas-Barceló I., Botet-Mussons F., Carbonell-Estrany X. (2020). Extrauterine growth restriction in very preterm infant: Etiology, diagnosis, and 2-year follow-up. Eur. J. Pediatr..

[32] Takahashi A., Bartolák-Suki E., Majumdar A., Suki B. (2015). Changes in respiratory elastance after deep inspirations reflect surface film functionality in mice with acute lung injury. J. Appl. Physiol..

[33] Smith L.C., Gow A.J., Abramova E., Vayas K., Guo C., Noto J., Lyman J., Rodriquez J., Gelfand-Titiyevskiy B., Malcolm C. (2023). Role of PPARγ in dyslipidemia and altered pulmonary functioning in mice following ozone exposure. Toxicol. Sci..

[34] Albertine K.H. (2013). Progress in understanding the pathogenesis of BPD using the baboon and sheep models. Semin. Perinatol..

[35] Cantu A., Gutierrez M.C., Dong X., Leek C., Sajti E., Lingappan K. (2023). Remarkable sex-specific differences at single-cell resolution in neonatal hyperoxic lung injury. Am. J. Physiol. Lung Cell Mol. Physiol..

[36] Grimm S.L., Dong X., Zhang Y., Carisey A.F., Arnold A.P., Moorthy B., Coarfa C., Lingappan K. (2021). Effect of sex chromosomes versus hormones in neonatal lung injury. JCI Insight..

[37] Fougère H., Bilodeau J.-F., Lavoie P.M., Mohamed I., Rudkowska I., Pronovost E., Simonyan D., Berthiaume L., Guillot M., Piedboeuf B. (2021). Docosahexaenoic acid-rich algae oil supplementation on breast milk fatty acid profile of mothers who delivered prematurely: A randomized clinical trial. Sci. Rep..

[38] Hellström A., Nilsson A.K., Wackernagel D., Pivodic A., Vanpee M., Sjöbom U., Hellgren G., Hallberg B., Domellöf M., Klevebro S. (2021). Effect of Enteral Lipid Supplement on Severe Retinopathy of Prematurity. JAMA Pediatr..

[39] Wendel K., Aas M.F., Gunnarsdottir G., Rossholt M.E., Bratlie M., Nordvik T., Landsend E.C.S., Fugelseth D., Domellöf M., Pripp A.H. (2023). Effect of arachidonic and docosahexaenoic acid supplementation on respiratory outcomes and neonatal morbidities in preterm infants. Clin. Nutr..

[40] McCulley D., Wienhold M., Sun X. (2015). The pulmonary mesenchyme directs lung development. Curr. Opin. Genet. Dev..

[41] Kyle J.E., Clair G., Bandyopadhyay G., Misra R.S., Zink E.M., Bloodsworth K.J., Shukla A.K., Du Y., Lillis J., Myers J.R. (2018). Cell type-resolved human lung lipidome reveals cellular cooperation in lung function. Sci. Rep..

[42] Varisco B.M., Ambalavanan N., Whitsett J.A., Hagood J.S. (2012). Hagood Thy-1 signals through PPARgamma to promote lipofibroblast differentiation in the developing lung. Am. J. Respir. Cell Mol. Biol..

[43] Scarpato M., Federico A., Ciccodicola A., Costa V. (2015). Novel transcription factor variants through RNA-sequencing: The importance of being “alternative”. Int. J. Mol. Sci..

[44] Kilroy G.E., Zhang X., Floyd Z.E. (2009). PPAR-gamma AF-2 domain functions as a component of a ubiquitin-dependent degradation signal. Obesity.

[45] Grygiel-Gorniak B. (2014). Peroxisome proliferator-activated receptors and their ligands: Nutritional and clinical implications—A review. Nutr. J..

[46] Torday J.S., Rehan V.K. (2006). Up-regulation of fetal rat lung parathyroid hormone-related protein gene regulatory network down-regulates the Sonic Hedgehog/Wnt/betacatenin gene regulatory network. Pediatr. Res..

