# Supplementary material for: Docosahexaenoic Acid Supplementation in Postnatal Growth Restricted Rats Does Not Normalize Lung Function or PPARγ Activity
Source: Biomolecules. 2025 Apr 9;15(4):551. doi: 10.3390/biom15040551 (PMC12024927; doi:10.3390/biom15040551)
Supplement: Supplementary file 1 [file biomolecules-15-00551-s001.zip › Supplementary Material.pdf]

<u>Supplementary Material</u>

<u>PPARγ and PPARγΔ5 Western Blots</u>

Optimal resolution of PPARγ and PPARγΔ5 on western blot requires different loading concentrations. Therefore, although both bands are visible on each blot, quantification of PPARγ was done on blot with 20μg total protein load, while PPARγΔ5 quantification was done on blot with 5μg 20μg total protein load. Male and female samples were performed on different blots. The corresponding uncropped Western Blot images and densitometry files are as follows:

Male:

<u>PPARγ Blot</u> – 57kB Band – File name: UNIVERSAL_02252025_161854_SuperSignalWestPicoPlus

<u>Total protein for normalization</u> – File name: UNIVERSAL_02252025_161854_No-StainLabeledMembrane

<u>PPARγ Densitometry</u> – 57kB Band (Band 2) File name: 2-26-2025_D21_Lung_Male

<u>PPARγΔ5 Blot</u> – 28kB Band – File name: UNIVERSAL_02252025_161854_SuperSignalWestPicoPlus

<u>Total protein for normalization</u> – File name: UNIVERSAL_02252025_161854_SuperSignalWestPicoPlus

<u>PPARγΔ5 Densitometry</u> – 28kB Band (Band 3) – File name: 57kB Band (Band 2) – File name: 2-25-2025_PPARg_Male

Female:

<u>PPARγ Blot</u> – 57kB Band – File name: UNIVERSAL_02262025_172846_SuperSignalWestPicoPlus

<u>Total protein for normalization</u> – File name: UNIVERSAL_02262025_172846_No-StainLabeledMembrane

<u>PPARγ Densitometry</u> – 57kB Band (Band 2) File name: 2-26-2025_D21_Lung_Female

<u>PPARγΔ5 Blot</u> – 28kB Band – File name: UNIVERSAL_02252025_162826_SuperSignalWestPicoPlus

<u>Total protein for normalization</u> – File name: UNIVERSAL_02252025_162826_No-StainLabeledMembrane

<u>PPARγΔ5 Densitometry</u> – 28kB Band (Band 3) – File name: 2-25-2025_PPARg_Females
