# Supplementary figures and images for: Docosahexaenoic Acid Supplementation in Postnatal Growth Restricted Rats Does Not Normalize Lung Function or PPARγ Activity
Source: Biomolecules. 2025 Apr 9;15(4):551. doi: 10.3390/biom15040551 (PMC12024927; doi:10.3390/biom15040551)

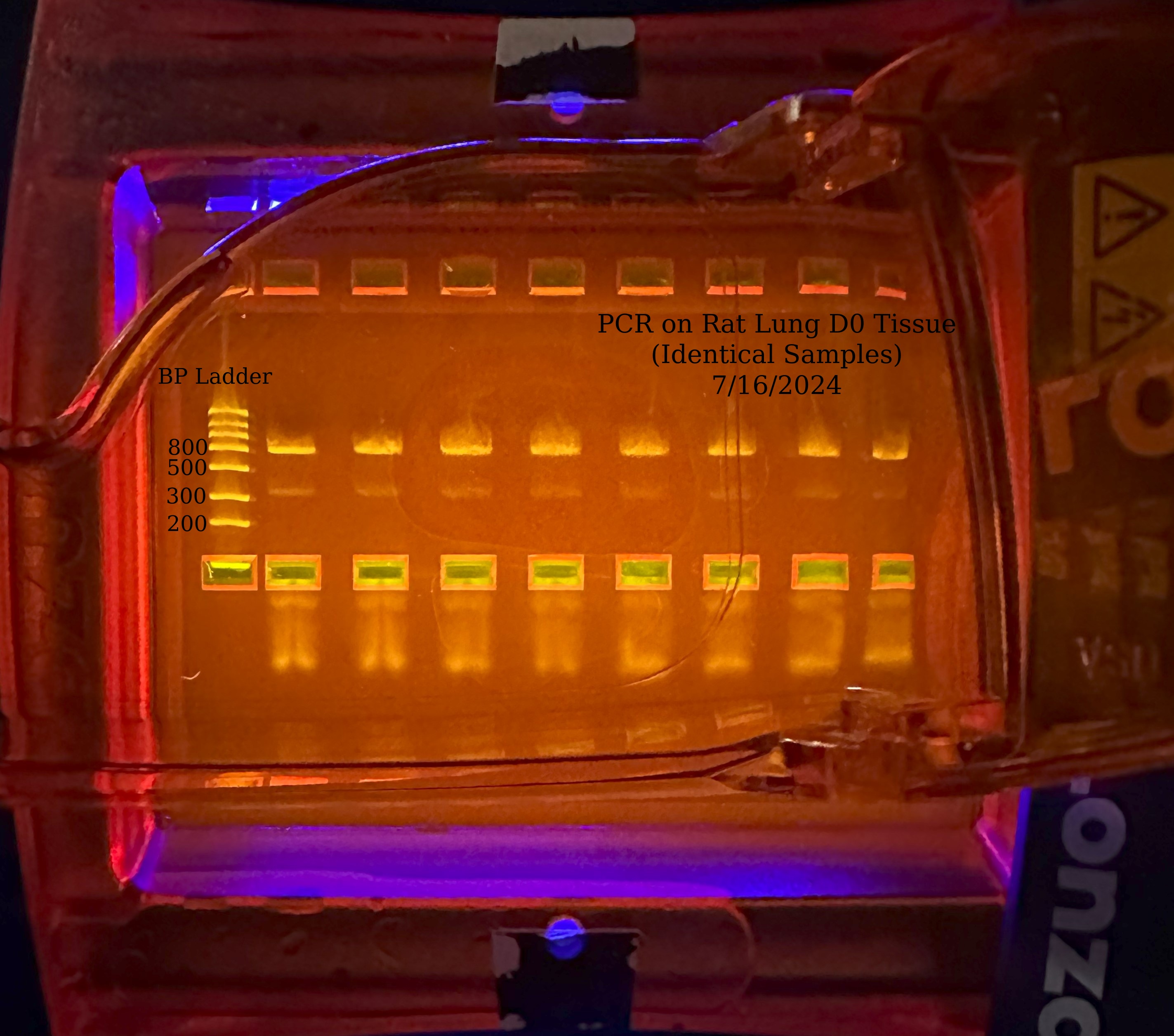

Supplement: Supplementary file 1 [file biomolecules-15-00551-s001.zip › Rat_Lung_PCR_Gel_Image.jpg]

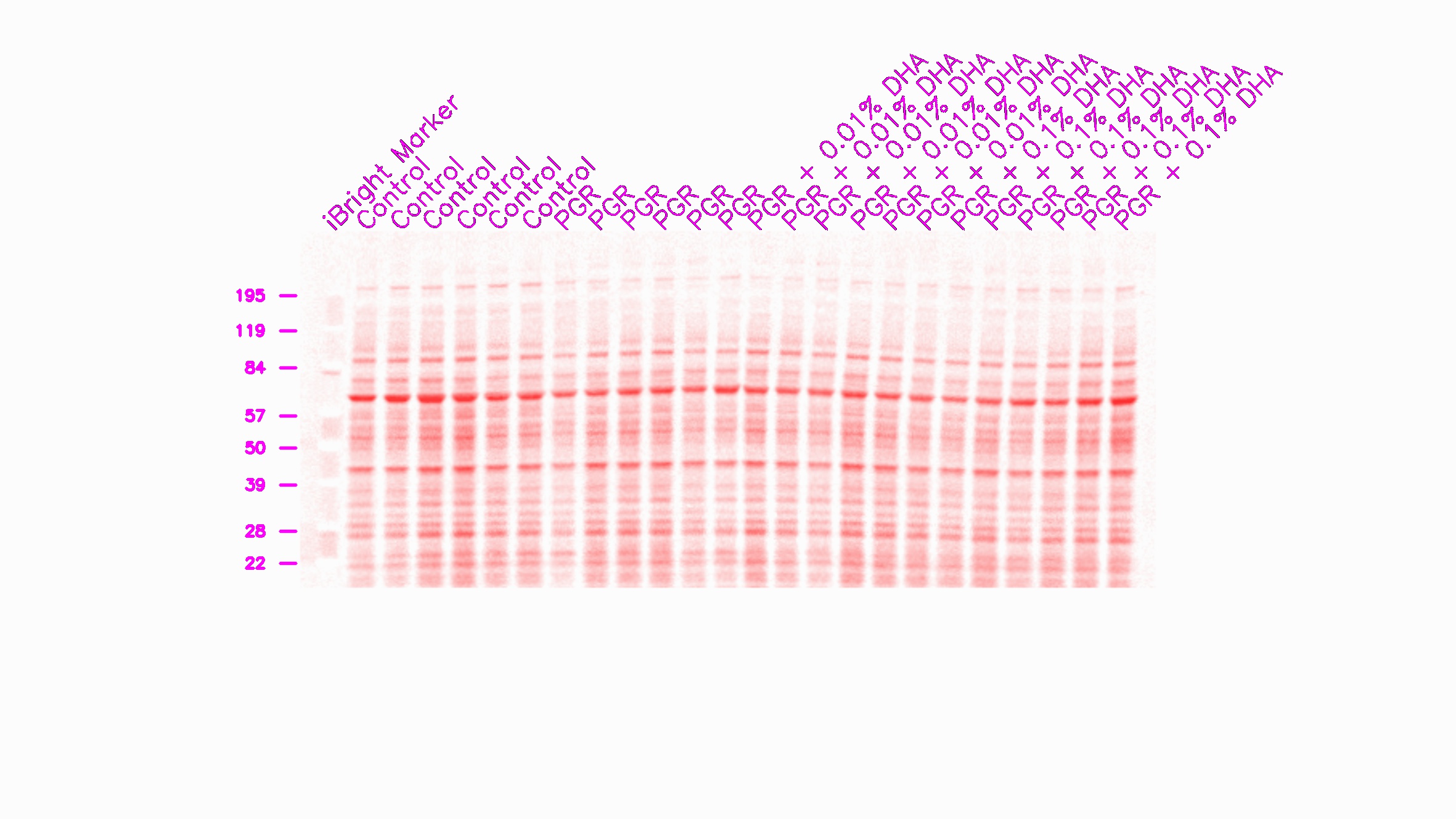

Supplement: Supplementary file 1 [file biomolecules-15-00551-s001.zip › UNIVERSAL_02252025_161854_No-StainLabeledMembrane.jpg]

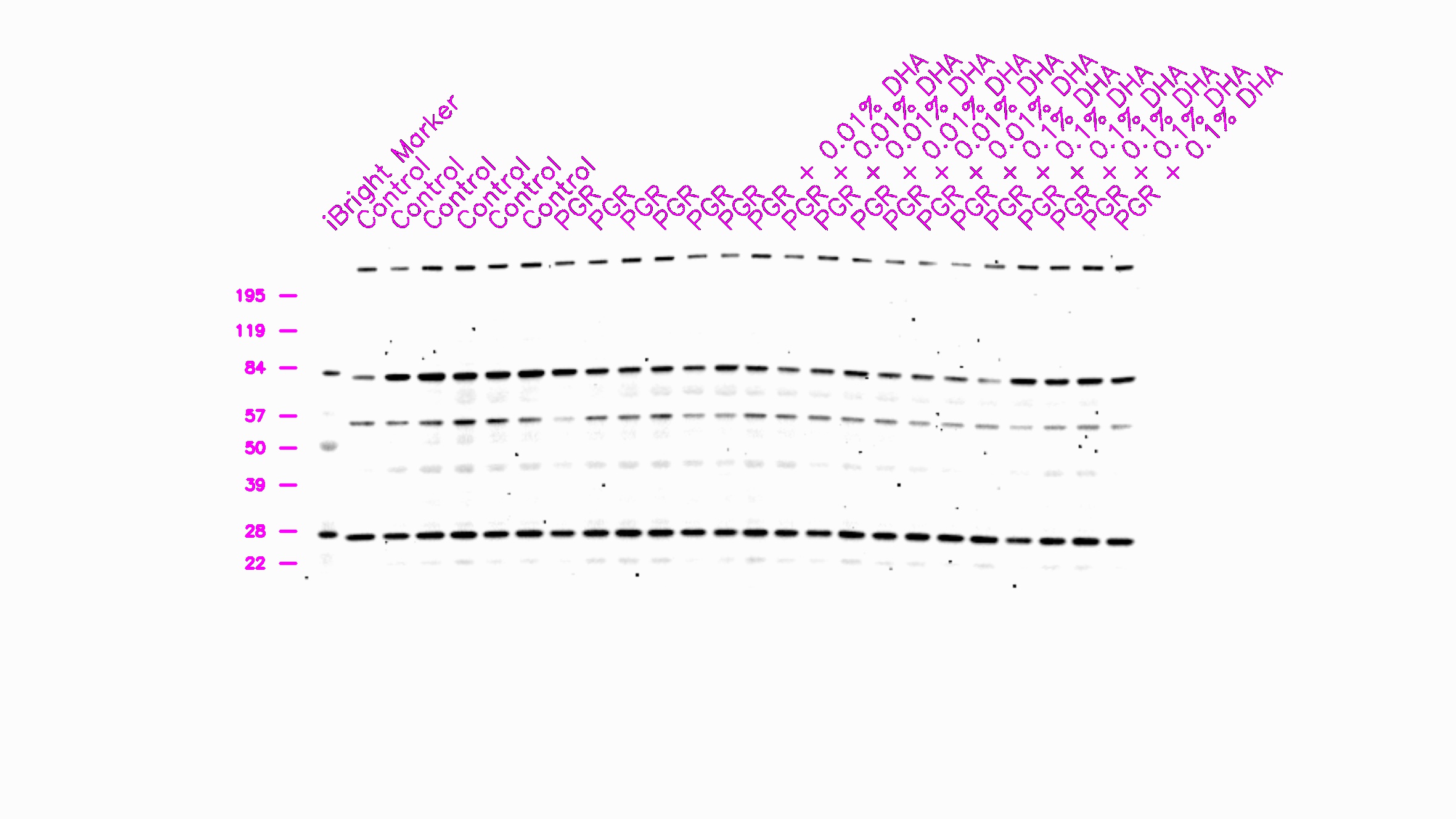

Supplement: Supplementary file 1 [file biomolecules-15-00551-s001.zip › UNIVERSAL_02252025_161854_SuperSignalWestPicoPlus.jpg]

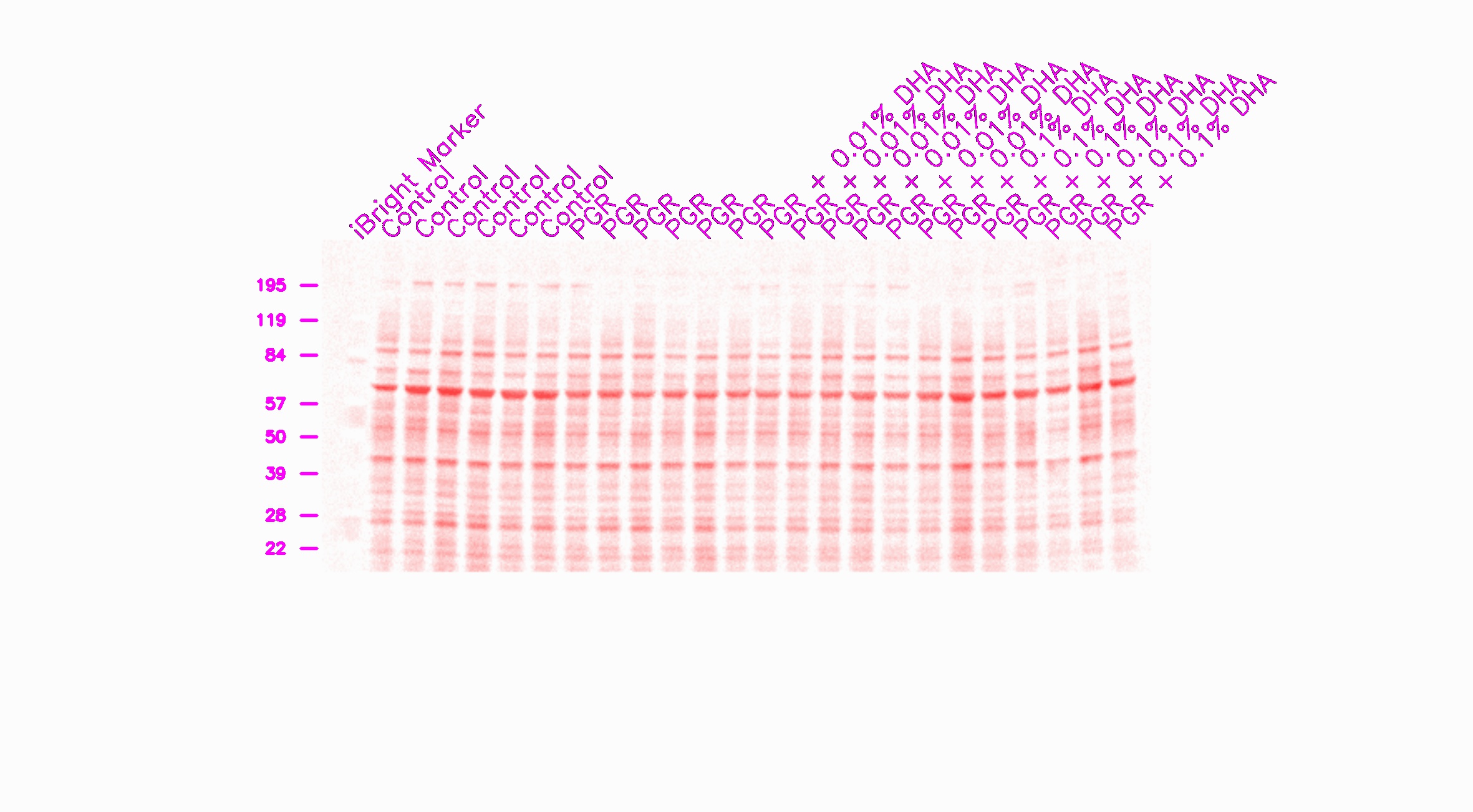

Supplement: Supplementary file 1 [file biomolecules-15-00551-s001.zip › UNIVERSAL_02252025_162826_No-StainLabeledMembrane.jpg]

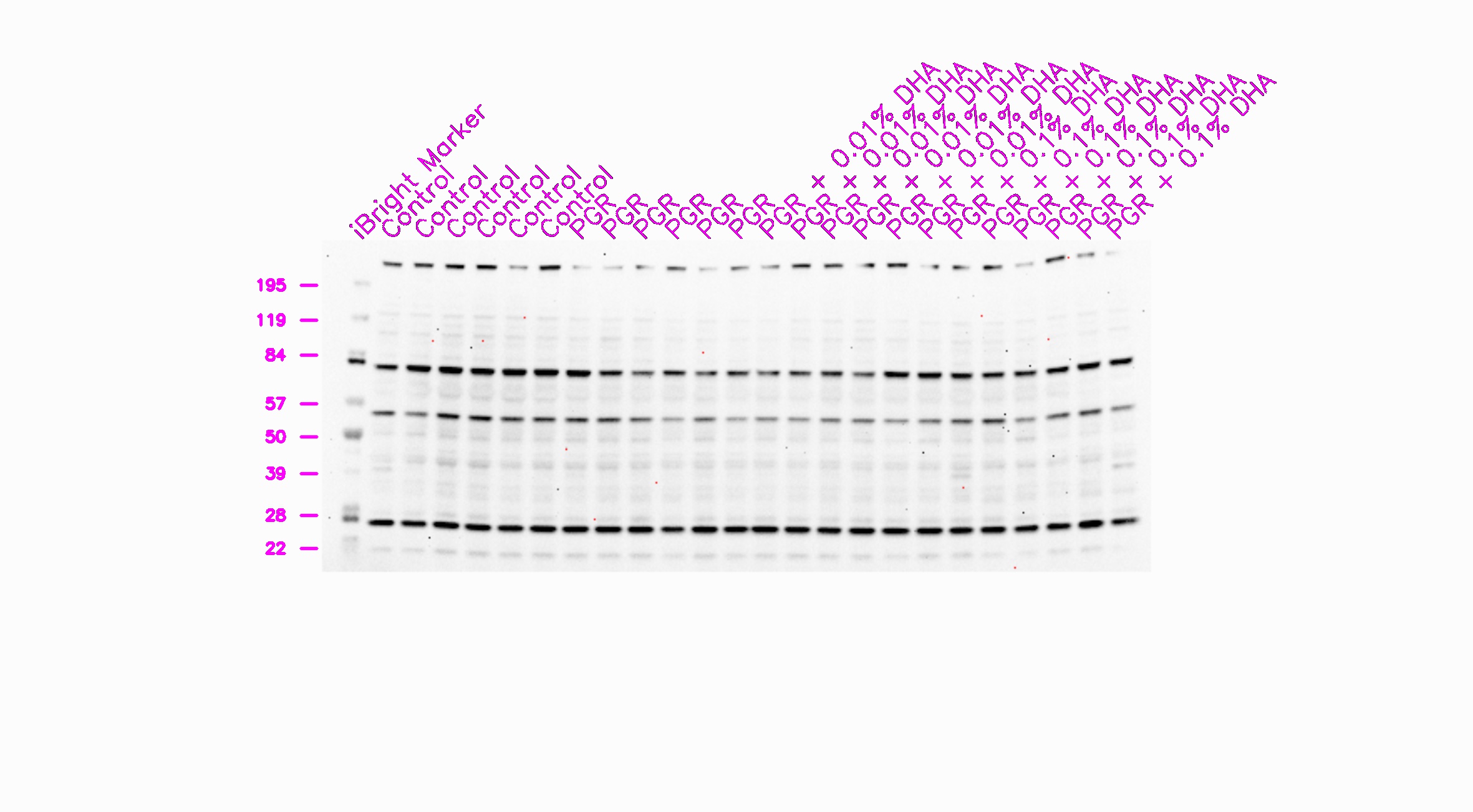

Supplement: Supplementary file 1 [file biomolecules-15-00551-s001.zip › UNIVERSAL_02252025_162826_SuperSignalWestPicoPlus.jpg]

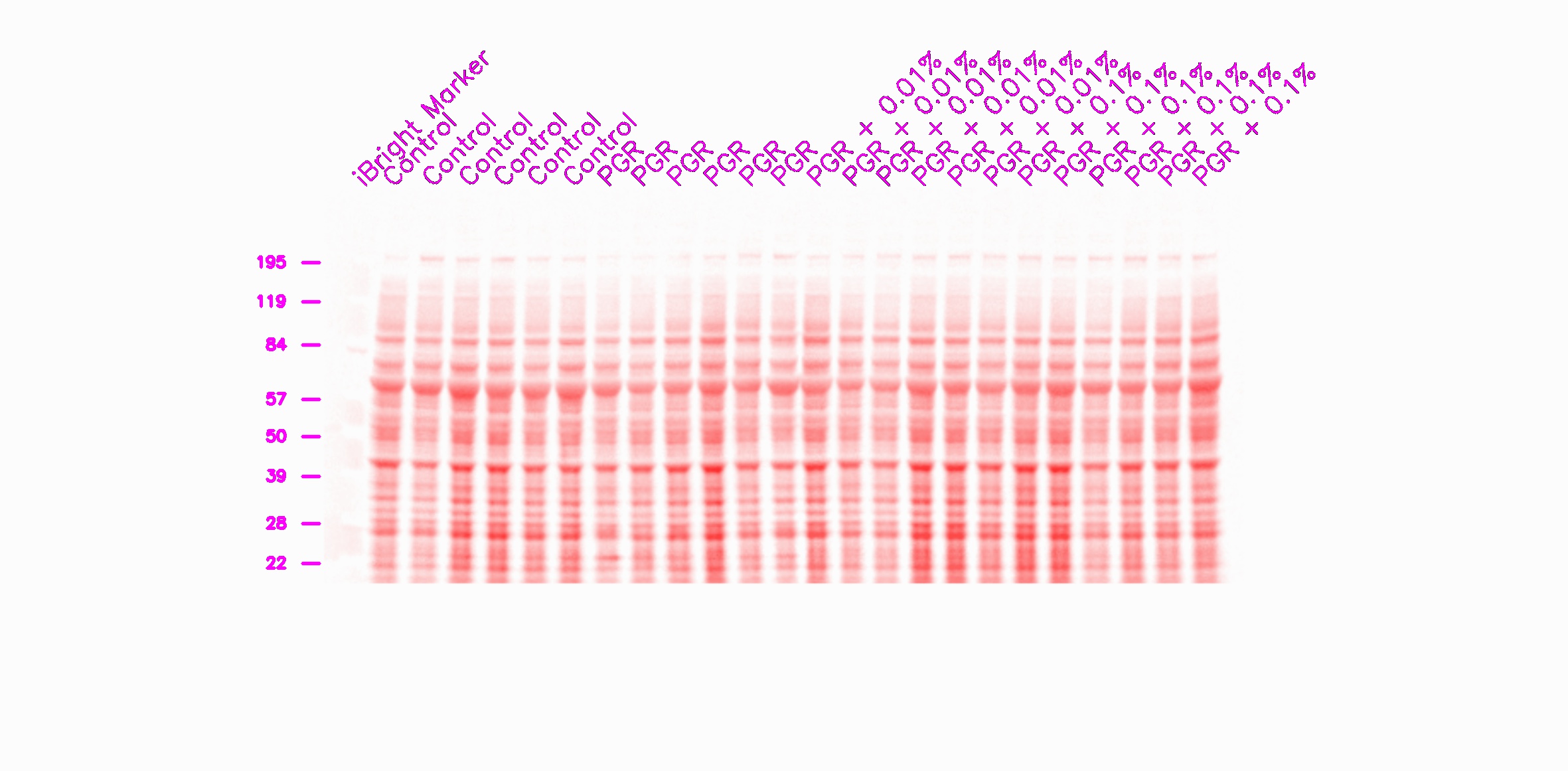

Supplement: Supplementary file 1 [file biomolecules-15-00551-s001.zip › UNIVERSAL_02262025_171754_No-StainLabeledMembrane.jpg]

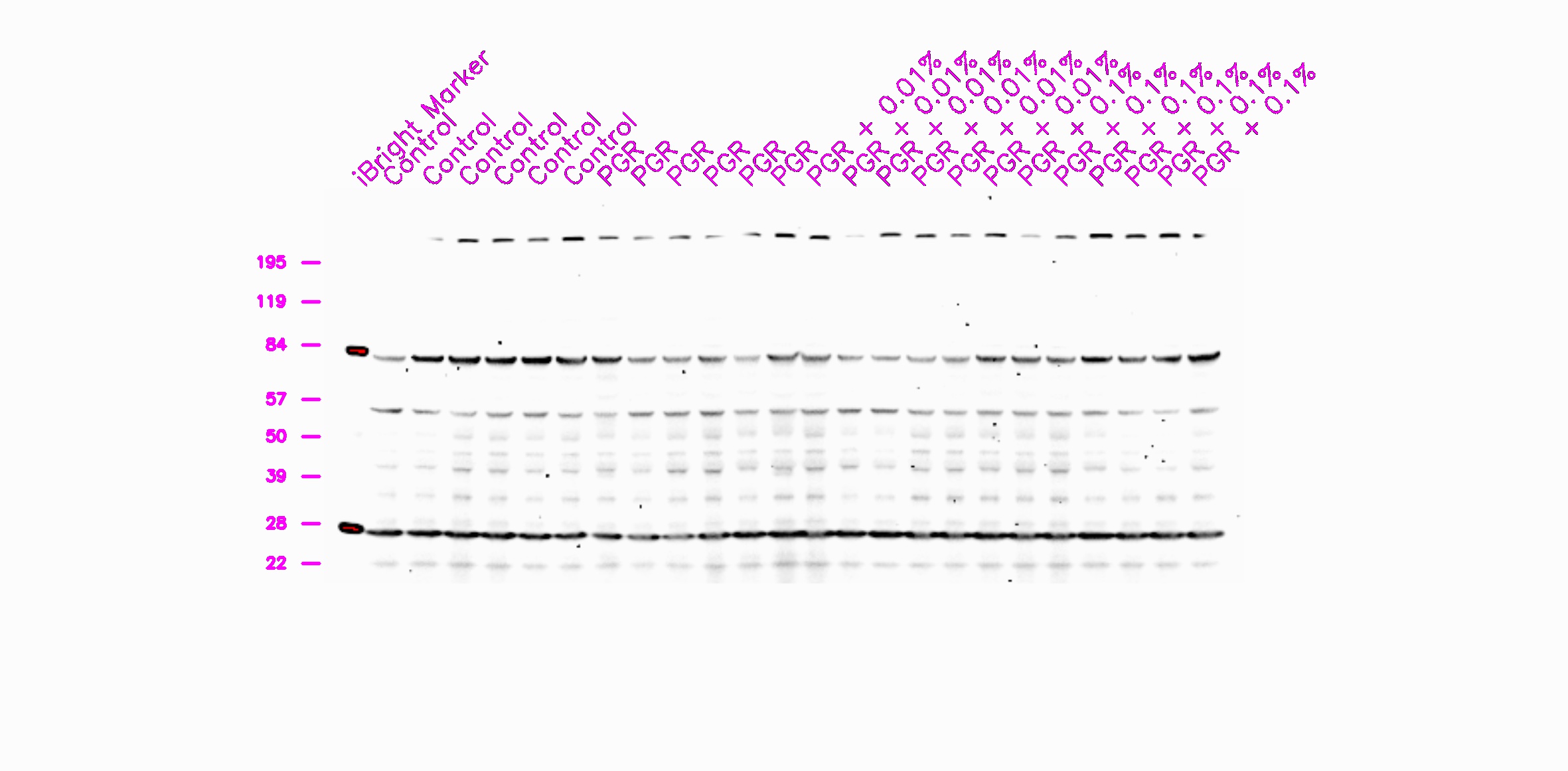

Supplement: Supplementary file 1 [file biomolecules-15-00551-s001.zip › UNIVERSAL_02262025_171754_SuperSignalWestPicoPlus.jpg]

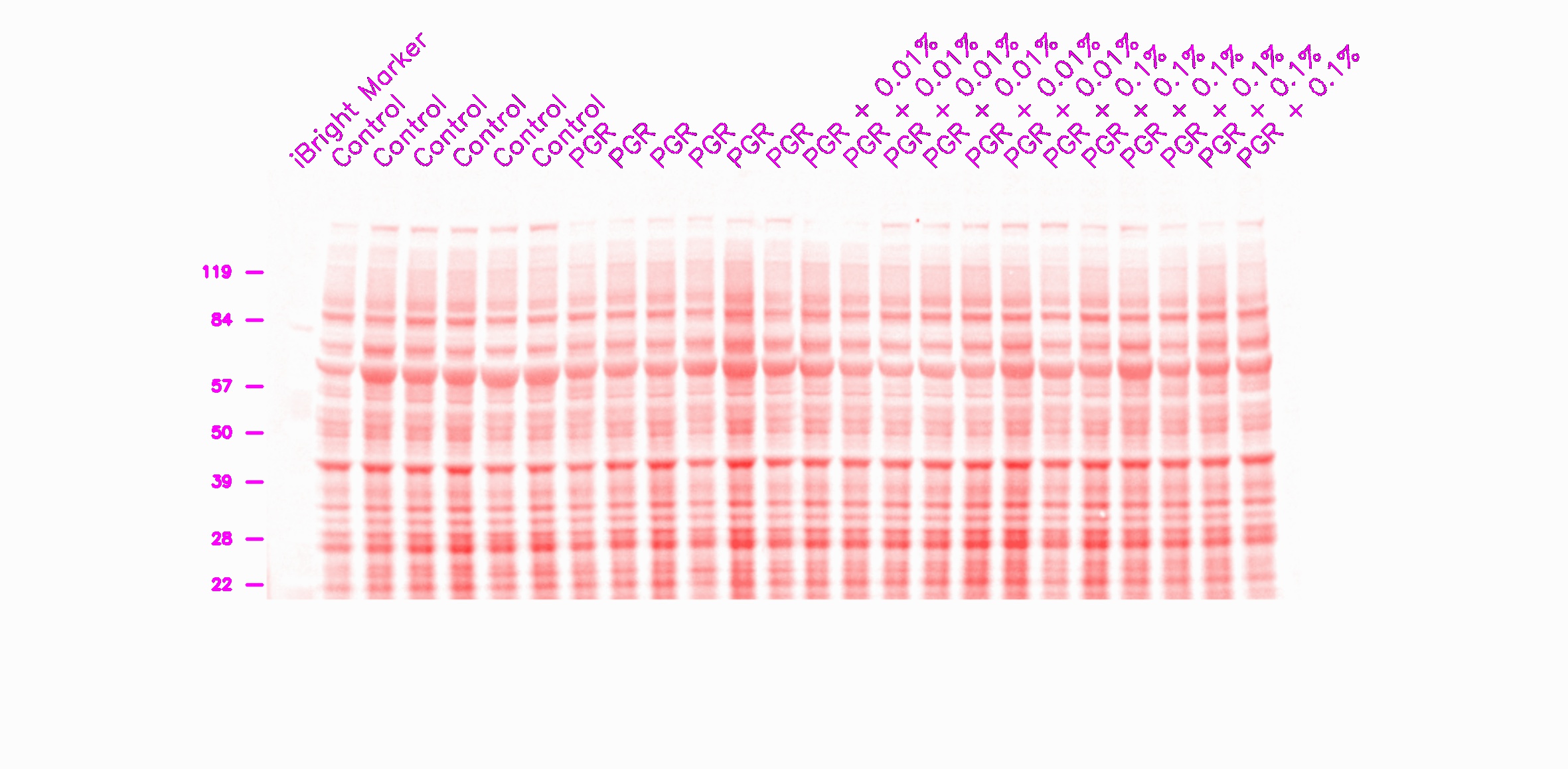

Supplement: Supplementary file 1 [file biomolecules-15-00551-s001.zip › UNIVERSAL_02262025_172846_No-StainLabeledMembrane.jpg]

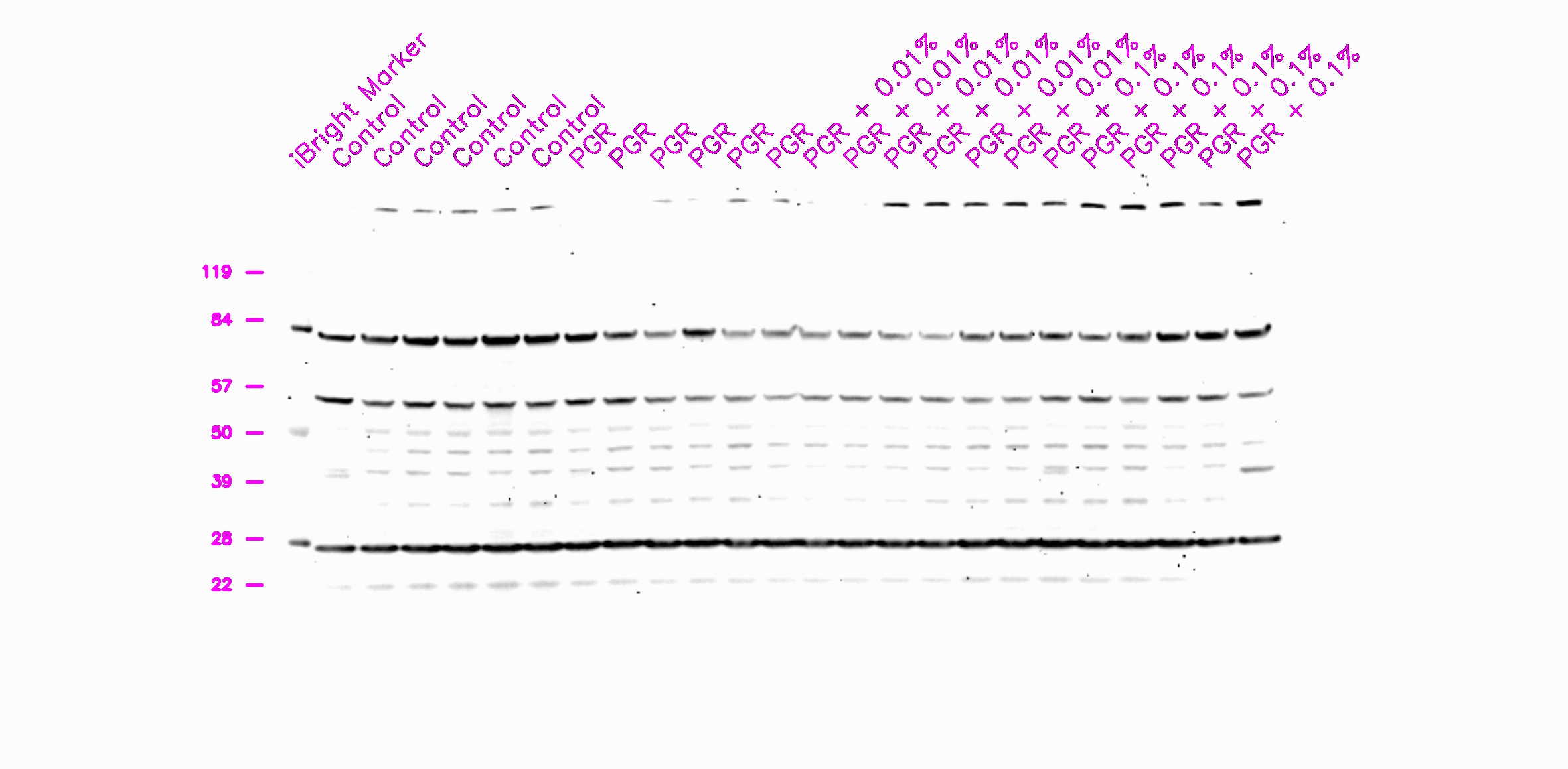

Supplement: Supplementary file 1 [file biomolecules-15-00551-s001.zip › UNIVERSAL_02262025_172846_SuperSignalWestPicoPlus.jpg]
